# Estimation of undernutrition and mean calorie intake in Africa: methodology, findings and implications

**DOI:** 10.1186/1476-072X-8-37

**Published:** 2009-06-27

**Authors:** Cornelia FA van Wesenbeeck, Michiel A Keyzer, Maarten Nubé

**Affiliations:** 1 Centre for World Food Studies, VU University, De Boelelaan 1105, 1081 HV Amsterdam, the Netherlands

## Abstract

**Background:**

As poverty and hunger are basic yardsticks of underdevelopment and destitution, the need for reliable statistics in this domain is self-evident. While the measurement of poverty through surveys is relatively well documented in the literature, for hunger, information is much scarcer, particularly for adults, and very different methodologies are applied for children and adults. Our paper seeks to improve on this practice in two ways. One is that we estimate the prevalence of undernutrition in sub-Saharan Africa (SSA) for both children and adults based on anthropometric data available at province or district level, and secondly, we estimate the mean calorie intake and implied calorie gap for SSA, also using anthropometric data on the same geographical aggregation level.

**Results:**

Our main results are, first, that we find a much lower prevalence of hunger than presented in the Millennium Development reports (17.3% against 27.8% for the continent as a whole). Secondly, we find that there is much less spread in mean calorie intake across the continent than reported by the Food and Agricultural Organization (FAO) in the State of Food and Agriculture, 2007, the only estimate that covers the whole of Africa. While FAO estimates for calorie availability vary from a low of 1760 Kcal/capita/day for Central Africa to a high of 2825 Kcal/capita/day for Southern Africa, our estimates lay in a range of 2245 Kcal/capita/day (Eastern Africa) to 2618 Kcal/capita/day for Southern Africa. Thirdly, we validate the main data sources used (the Demographic and Health Surveys) by comparing them over time and with other available data sources for various countries.

**Conclusion:**

We conclude that the picture of Africa that emerges from anthropometric data is much less negative than that usually presented. Especially for Eastern and Central Africa, the nutritional status is less critical than commonly assumed and also mean calorie intake is higher, which implies that agricultural production and hence income must also have been growing at a pace at least high enough to keep up with population growth. In terms of methodology, our estimates form a base line for 2005 for the whole continent that can be easily updated with far less information for individual countries, as we show in an example for Ethiopia.

## Background

The international debate on strategies to fight poverty and hunger very much relies on headcounts of people below a certain poverty line and people below given nutritional standards. However, the evidence and methodology these estimates are based on differ much between both yardsticks.

For poverty, several agencies have for many years been engaged on a regular basis in collection and compilation of household surveys in support of progress at national and regional level. Trends are often reported on through poverty maps, and, in addition, a poverty gap is estimated as indicative of the resources needed to bring the entire population above the defined poverty line. On the methodological front, the main challenge is to combine these survey data with other sources such as population density maps and census information, to obtain estimates with a national coverage [1-3].

For estimates of the nutritional status of people, the situation is less straightforward. Even though many, usually small-scale surveys have been undertaken for specialized research, few sources are accessible that provide internationally comparable data on nutritional status. In fact, for Africa, such data are available only for young children and women of reproductive age in the Demographic and Health Surveys (DHS, commissioned by the United States Agency for International Development, USAID). These surveys have a nationwide coverage, and are widely used to assess the nutritional status and development of children and women, amongst others by the United Nations Children's' Fund (UNICEF), and the World Health Organization (WHO), but not that of men [4,5]. For national headcounts of undernutrition the international community relies on an indirect measure developed by the Food and Agricultural Organization of the UN (FAO) [6]. This measure is obtained by distributing food availability, measured as production plus net import at national level, among household groups on the basis of information from household expenditure surveys. This yields food availability by household group, and comparison with a nutrition standard, a minimum calorie intake, leads to the gap, from which the headcount can be derived. The method has been criticized for being unnecessarily indirect and sensitive to assumptions [7-9]. In addition, its headcount and its food gap are computed at national level only. Consequently, it is unable to pinpoint the "hunger hot spots" where policy interventions, possibly including food aid, may be required, and equally unable to relate the problem to local conditions.

A recent study by the International Food Policy Research Institute (IFPRI) has used Household Expenditure Surveys for the estimation of the prevalence of hunger and the food gap [10]. Based on data for 12 countries in sub-Saharan Africa (SSA), IFPRI concludes that their estimates of hunger are more closely correlated with measures of poverty than those presented by FAO. While FAO's estimates are based on national food production estimates (supplemented with information on imports, withdrawals from stocks, etc and distribution of expenditures), the Household Expenditure Surveys used by IFPRI provide direct estimates of household food consumption on the basis of actual amounts of food (bought and home produced). With respect to national per capita food consumption differences are pronounced, but of varying signs across countries. Although the direct use of anthropometric data as a measurement of wellbeing is widely spread (especially data on children with respect to weight-for-age, height-for-age, and weight-for-height), comparing anthropometric data with other measures for poverty is much less common. In their study on alternative measures of welfare, Sahn and Stifel use data on child health and nutrition to validate a new approach to measure income via assets and find that an index of assets indeed outperforms expenditure data in its correlation with these indicators and hence advocate the use of such an index as a means of mapping economic data to information on health and nutrition [11]. For the Arab region, Boutayeb and Serghini also find a high correlation between data on health indicators and human development [12]. Ebener et al. take the correlation between wealth and health indicators as given and propose to use satellite images of night-time light to arrive at sub-national estimates of wealth that in turn lead to sub-national estimates of health [13]. A more common link is that between nutrition and poverty, e.g. through the definition of poverty lines, among other things used to provide poverty maps, spatially specific overviews of the prevalence of poverty. Following Ravallion, the "food" poverty line is the level of expenditures that is needed to obtain a certain minimum amount of calories, usually 2100 Kcal per person per day at household level [14]. An obvious problem in measuring poverty this way is that this minimum amount of calories can be reached by very diverse diets, with different implications for costs [15]. A less obvious issue is how much calories are actually needed to ensure an intake of 2100 Kcal per person on average in the household as household waste and processing losses are difficult to estimate. This also implies that a reverse inference (what is the nutritional status of the poor given their income and expenditure) may be very difficult and requires much more information than usually contained in household expenditure surveys.

The lack of data and the differences in outcomes obtained from various methods make it all the more essential to make full use of all information available. In this connection, this paper aims at making two contributions: first, we develop for the year 2005 an estimate of the total number of people suffering from undernutrition in sub-Saharan Africa, based on anthropometric information available for women and children in the DHS surveys at province and district level. These surveys cover 48 of the 50 countries in SSA. In our estimate for the year 2005, thirty-three DHS surveys were used, complemented with data from WHO. Our methodology for estimating undernutrition among adults closely follows the generally accepted method to estimate this number for children. It needs some additional assumptions though, because data are scarce or lacking for males of all ages and for adults in general for particular age brackets, or for particular countries. Yet, we provide evidence suggesting that our estimates for adult undernutrition are not much less reliable than the widely accepted estimates for underweight children.

The second contribution of the paper is to estimate mean calorie intake at sub-national level, based on recorded weights of women and children in the same surveys. Since the DHS data are usually reported at the district or province level, our estimate of the food gap is at the same geographical level and hence far more explicit than the national totals presented by FAO [6]. Furthermore, we show how the estimates of calorie intake also provide some link between nutritional science and economics, since all that is consumed must either be imported, or produced within the country itself. In this respect, we reverse the methodology followed by FAO, in that we infer agricultural production (and hence, agricultural income) from anthropometrically-based consumption estimates.

Since our methodology relies so much on anthropometric data taken from DHS surveys, we also include a discussion on the quality and representativeness of these surveys, an exercise that, to our knowledge, has not been done before, despite the widespread use of this source for the monitoring of children's nutrition and health.

The paper proceeds as follows. Section "Methods" describes our methodology to estimate undernutrition and calorie intake on the basis of anthropometry. Section "Results" presents our estimates for undernutrition and calorie intake. Section "Discussion" focuses on critical steps in the estimation procedure, presents a discussion on the reliability of the main source of data (the DHS surveys), compares our results to other studies, sketches implications for the economic conditions in SSA and describes a procedure to enhance the policy relevance of the estimates, applied to the situation in 2008 in Ethiopia. General conclusions are summarized in the final Section "Conclusions".

## Methods

### Estimating undernutrition prevalence in sub-Sahara Africa on the basis of anthropometry

When all necessary anthropometric data are available, estimation of undernutrition is relatively straightforward but this is seldom the case. As a rule, various assumptions have to be made to fill data gaps, especially for males, adolescents and the elderly as DHS surveys only report on the nutritional status of children and adult women. For children, we use the new growth reference data issued by WHO, which are based on new growth studies in children from well-to-do families in various parts of the world, and hence use the reinterpretation of the data in the DHS surveys by WHO [5].

For children under five, we use the results from the DHS-surveys on the prevalence rates of children with a low weight-for-age (2standard deviations below norm weight). For this group, in some surveys data are only available for children of 0–3 years instead of 0–5 years. For countries where this is the case, the underweight prevalence rate in the 0–3 years group has been used, as e.g. Nubé and Sonneveld found that the ratio of the percentages underweight children aged under 5 years to aged under 3 years is generally very close to one [16].

For children 5–10 years of age we take underweight prevalence to be equal to the 0–5 years age group. Few anthropometric data are available to substantiate this assumption, but for four African countries (Madagascar, Ethiopia, Zimbabwe and Lesotho), the underweight prevalence rates in the 0–5 years and the 5–10 years age group have been reported to be rather similar [17-19].

With respect to the undernutrition prevalence in the 10–15 age group, there is no universally accepted method for the measurement of undernutrition [20-22]. Most commonly used indicators are thinness, measured as the percentage of the population with a BMI-for age (Body Mass Index = weight in kilograms divided by the square height in meters) below the 5th percentile of a norm BMI-for-age, and stunting, measured as the percentage of the population with a height-for-age below the 3rd percentile of a norm height-for-age. Recently, WHO has revised its growth reference for the age group 5–19 years, with the objective to bring the growth standards in accordance with the new WHO Child Growth Standards (0–5 years) [5,23,24]. Both indicators may yield significantly different results, also varying by age, largely because adolescence is a period of rapid growth and rapid changing body composition. As no representative data on undernutrition among the 10–15 year age group are available (DHS-surveys do not cover this age segment), we take the undernutrition prevalence in the 10–15 years age group of a particular country to be the same as the one in the 15–20 segment reported in DHS-surveys.

Next, for the age bracket of 15 years and older, we rely on the measurements for women from the DHS-surveys, supposing that there are no major differences in nutritional status between males and females, except for the countries in Southern Africa, since for South Africa and Swaziland, whose DHS surveys separately record men's weights, the nutritional status turns out to be worse for men, a finding that was also confirmed in personal communication with Joyce Luma, Chief Vulnerability Analysis and Mapping Unit, WFP, Rome. For other regions of sub-Saharan Africa the DHS surveys do not record the nutritional status of adult males, and such data are generally quite scarce. Yet, on the basis of a limited number of small-scale studies, Nubé and Van den Boom argued that for West, East and Central African countries the difference between prevalence of underweight among adult males and adult females tends to be very small: in terms of mean BMI, males are about one point below the females [25]. Yet, we consider the available data to be too limited to warrant the assumption of a lower BMI for men in these three regions.

For elderly people, we use the undernutrition prevalence rates as reported for adults. This might cause some underestimation of the total number of persons suffering from undernutrition, as the tendency has been reported of a less favourable nutritional status in this age group [26,27]. Nonetheless, we find the empirical evidence insufficient to justify a separate prevalence rate. We may note that in this class the less favourable condition generally does not develop before the age of 60, which in particular in low income countries only constitutes a small segment of the population.

Finally, for countries without any complete and relatively recent DHS survey or other reliable source of information on undernutrition prevalence, we impute the missing data by borrowing from countries that are close in the Human Development Indicator for 2005. Countries that are closest in general living standard are identified, and the undernutrition prevalence rates of these countries are applied. The only exception to this rule in Angola, where we used the WFP analysis of the food security situation as source for the nutritional status of women [28]. The full list of inferences made and procedures applied is included separately [see Additional file 1].

Table 1 lists the other sources of data used, in this study, both for estimation of undernutrition discussed here and for the estimation of calorie intake to be discussed in the next section.

**Table 1:** Overview of data sources other than DHS survey reports

	**Survey reports**	**Census**	**Technical**
			**coefficients**
**Data set**	WHO reports on child	Population tables	Human Energy
	nutritional status, from		Requirements
	DHS surveys		
**Origin**	WHO/USAID	Population	FAO/WHO/UNU
		Division UN	Expert consultation
			2001
**Country**	46 countries	48 countries	----
**coverage**			
**Time series**	1995, 1996, 1998, 1999, 2000, 2001, 2003, 2004, 2005, 2006, 2007	1985, 1990, 1995, 2000, 2005	-----
**Variables**	Percentage of underweight and severely underweight children (2006 revision)	Population numbers by sex and age	Calorie needs by sex and age related to weight and PAL factors

### Estimating calorie intake in Africa

Having argued and documented that direct measurement of undernutrition in children and adult women can offer a relatively reliable basis for estimating undernutrition among the overall population, we are now ready to move to the next step, which is to estimate average calorie intake among the population, and also the prevailing calorie deficits.

This obviously is not an aim in itself. The anthropometric measures discussed so far only describe the end result of the nutrition chain. To understand how this result came about it may be useful to track it back to the information on food production and distribution. This makes it possible to assess the trends in terms of economic performance, growth and development. In addition, by tracing the demand implications of a particular anthropometric status by subregion and age group and by sex, it becomes possible to build up estimates of food deficits from its constituent parts, with possible implications for food aid needs. Finally, calorie consumption plays a pivotal role in FAO's estimates of undernutrition and comparing our findings on this variable may reveal causes of differences in estimates of undernutrition prevalence.

Clearly, the procedure will involve several steps, from anthropometry – in particular actual weight – via physical activity level of the individuals concerned, to their nutritional intake that is biophysically associated to this weight. We calculate in parallel the nutritional intake needed under the prevailing activity level to provide adequate nutrition and from this obtain the food deficit of a particular group. Summation over the population groups yields the average calorie intake as well as the overall food deficit.

Hence, the weight and height of individuals as recorded in the Demographic and Health Surveys (DHS) provide the anchor for our estimation of calorie intake. In addition, we use anthropometric data from other sources. The basic nutritional insight enabling us to establish the link between anthropometry and calorie demand is that there exists a strong biophysical relation between the weight of a person and the calorie intake that is needed to sustain this weight. This relation is usually invoked only to determine a recommended level of calorie intake but our estimation of actual calorie intake uses it in reverse direction: if a person has a certain weight, then this implies that the calorie intake must have sustained this weight during the period preceding the measurement, given specific allowances for physical activity (adults in general), for growth (children), and for pregnancy and lactation (women).

For all age groups and for both sexes, FAO estimates and publishes the relations between weights and calorie intake, with physical activity levels and birth rates as parameters [29]. The general structure of the relations is as follows (for notational convenience, a regional subscript is omitted):

(1)

where the subscript *g *denotes gender and *t *denotes age; *cal*_*g*, *t *_is the calculated daily calorie intake by age and gender, *w*_*g*, *t *_is the measured weight of the person, *G*_*g*, *t *_is a parameter representingallowance for growth; *A*_*g*, *t*_, *b*_*g*, *t*_, *c*_*g*, *t *_are other given parameters, *PAL*_*g*, *t *_is the Physical Activity Level correction factor, and *R*_*g*, *t *_is the birth rate by age group for women in fertile age groups. Hence, all coefficients are gender- and age-specific.

National figures for birth rates are obtained from FAO, and supposed to be equal for all women in the fertile age groups [29]. This is a simplification, of course, but it may be warranted as we only use the figures to calculate nutritional requirements of mothers, as opposed to the births themselves. Growth allowances and values of the parameters *A*_*g*, *t*_, *b*_*g*, *t*_, *c*_*g*, *t *_are taken from FAO [30].

The survey information used contain data on the weight of women, as well as on the percentage of children aged 0–5 (or 0–3, see the previous section) that are underweight, measured as having a weight below 2 sd of the norm for their sex and age group. Hence, whereas for women weights are observed directly, for children we only have data on the percentages of children from 0 to 5 years of age that are underweight (2 sd below norm weight) and severely underweight (3 sd below norm weight). For this age group we calculate the average weight as follows:

(2)

where *w*_*t*, *g *_is the calculated average weight of the child, *sd*_2_, and *sd*_3 _are the shares of children with weight below 2, respectively below 3 standard deviations of the norm weight for their age, and , , and  are the norm weight, the weight at 2 sd below norm and the weight at 3 sd below norm, respectively. We note that as these norm weights follow established international standards they are the same for all countries. The factor *β*, the ratio of the average BMI of the women in the area over the norm of 18.5, adjusts these weights assuming the nutritional status of children that are not underweight to follow that of the mother. For the age group 5–9 years, as in the estimation of undernutrition prevalence, we assume similar rates of undernutrition prevalence as they exist for the 0–5 years age group, and use the same procedure as for the age group 0–5. For female adolescents of ages 10–14 years, weights are based on women of ages 15–19 years, on the basis of the ratios between weights at different ages from FAO [29,30].

For men, we use FAO estimates of weights of women and men of all age groups for all countries, and apply the ratio of the weight of males and females in the same age group to calculate the weight of the men [29]. Hence as before, we abstract from any major differences between men and women as far as nutritional status is concerned. Recalling that in sub-Sahara Africa the nutritional status of females might be somewhat better than that of males; this might result in a slight overestimate of kcal consumption. Since for South Africa and Swaziland, data for men are available directly from the DHS surveys, for these countries, direct estimates of the nutritional status of men are made, and the relative status of men versus women in these countries is used to infer the status of men for the whole of Southern Africa. For the other regions, following Nubé, no correction for the possible difference between men and women was made in view of the lack of reliable large-scale studies on possible differences between adult male and female nutritional status [31].

Finally, since country surveys are not always available for the common reference year 2005, inference is required and our estimates for energy consumption suppose that there are no major variations within the time span of a few years, in body weight for people within the same age bracket. This assumption is supported by anthropometric data from countries where two or more DHS-surveys have been implemented over the past 10–15 years. In most of these countries, the mean weight of the adult population changes by, at most, 2–3 kg over periods of around 5 years. The amounts of energy (calories) implicated in such changes are in the order of magnitude of 10–15 kcal per day only, and hence remain well within the one percent range for a total per capita energy expenditure of around 1800–2200 kcal.

Another major factor affecting the estimation of per capita consumption by gender and age is the correction for physical activity. The PAL (Physical Activity Level) is defined as the ratio of the Total Energy Expenditure (TEE) to the Basal Metabolic Rate (BMR) [29,30]. The minimal correction is a factor 1.58 that corresponds to very light activity such as sleeping, sitting, and standing, and increases with the activity level. Our calculations apply separate PAL values for urban and rural consumers, and take as point of departure 1.58 for urban and 1.8 for rural consumers, following FAO, in which also data on the percentage of the population in a country that is labelled "urban" is provided [29]. To account for possible differences in estimates of urban and rural population, we corrected the PAL as follows: if the share of urban consumers in total is higher than that reported by FAO, we conclude that in our database the urban areas also include population that the FAO classifies as rural. In this case we increased the PAL factor for the urban consumers relative to the level reported in FAO according to:

(3)

Where  is the urban population implied by FAO, *POP*_*u *_is the urban population in our own database, and 1.58 and 1.8 are the PAL factors for urban and rural areas, respectively, as given in FAO for all countries considered here. For rural areas, we start from the PAL factor of 1.8 for all rural consumers as reported by FAO and correct this value downwards if the bodyweight is low, to reflect the fact that malnourished people will tend to adjust their activity level [32].

There are various methods for empirically assessing the total energy expenditure of individuals or groups of individuals. The traditional ones include collection of detailed information on total daily food consumption by households say, from food expenditure surveys, followed by a conversion of the various foods into calories. This approach tends to suffer from high error margins because of poor recording of both the acquisition of food and the food intake during meals. Two other methods for estimating total energy expenditure are generally considered to yield reliable results. The first, and probably most reliable method, is the measurement of total energy expenditure with the Doubly Labelled Water method (DLW) [30]. The second method, also increasingly applied, is the Heart Rate Monitoring method (HR) [33]. While both the DLW- and HR-methods are mainly applied in research in industrialized countries, some results have been published for developing countries but mainly in Asia. These studies report for rural settings PAL-values in the order of 1.8–1.9, whereas our maximum PAL-value for rural areas is set at 1.8 [32,34,35]. For urban settings, the reported PAL values are in the order of 1.6–1.8, which is also higher than the PAL-value of 1.58 used here for fully urbanized population groups [32,36-38]. It should be mentioned that most of these studies limit their measurements to small samples of persons. Nonetheless, when compared to the data available for developing countries our assumed PAL-values fall in the lower end of the range, indicating that our estimates of calorie consumption may be on the conservative side.

The next step in the estimation of total calorie consumption is the calculation of rural, urban, and national average calorie intake. For this, we use data on the structure of the population by age group and gender, as published by the United Nations Population Division [39-42].

Finally, to allow comparison with estimates as presented e.g. by FAO and IFPRI, we must make a final step from *calorie intake *to food availability. At household level, in high income countries the total consumption that does not result in human intake runs into the 300–400 kcal per person per day [43-46]. Losses at retail level and in institutional feeding are probably larger, but reliable data are not available. As a conservative estimate, we estimate the food wastes at 200 and 100 kcal per capita per day in urban and rural areas, respectively, with a maximum of 10 and 5 per cent, respectively, in total per capita consumption.

For countries where no complete, recent, DHS survey is available, we impute the missing data as described in the previous section. The full list of inferences made and procedures used is included separately [see Additional file 1].

## Results

### Estimates of undernutrition

**Figure 1:**
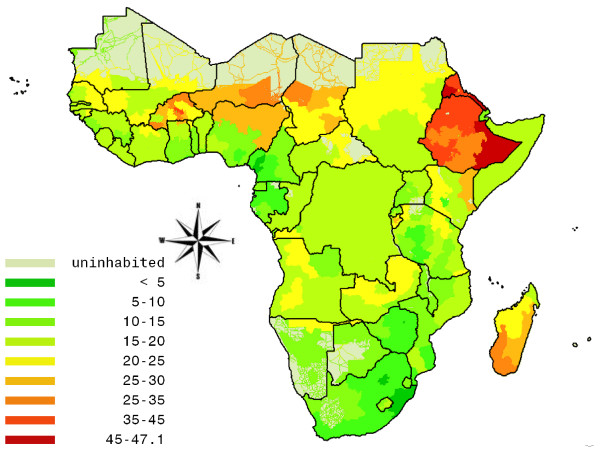
**Undernutrition map of sub-Saharan Africa: % of total population in 2005 that suffers from undernutrition**. Source: own calculations.

For the year 2005, we estimate that 131 million people or 17.3% of the total population in SSA (all age groups combined) suffer from undernutrition. Southern Africa clearly performs best, with only 3 million people (5.9% of total population) being classified as suffering from undernutrition. The other regions (Western Africa: 16.3%, Central Africa: 18.2%, Eastern Africa: 19.6%) are all relatively similar with respect to the average nutritional status of their populations, although large differences within these regions, and even within countries exist, as is illustrated in Figure 1. The figure depicts the percentage of the population suffering from undernutrition in sub-Saharan Africa as a whole, where clear geographical patterns of hunger can be seen, with marked hotspots in Ethiopia, and Eritrea, but also in the relatively well-off region of West-Africa (e.g. areas of Burkina Faso)

### Estimates of calorie intake

We estimate the average daily per capita calorie intake for sub-Saharan Africa to be 2098 Kcal/capita/day, with region-specific estimates of 2120 Kcal/capita/day for Western Africa, 2041 for Central Africa; 2045 for Eastern Africa and 2418 for Southern Africa.

**Figure 2:**
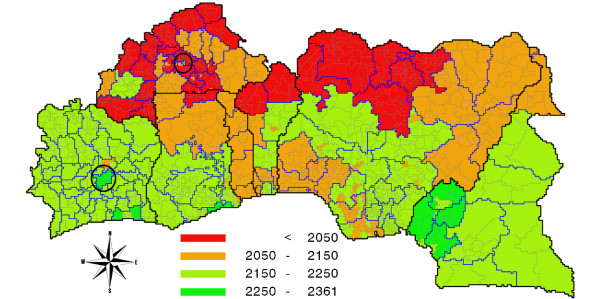
**Nutrition map of West Africa: per capita daily food intake in 2005 in kilocalories**. Source: own calculations.

As an illustration of the geographical spread of per capita consumption across the continent at a less aggregate level, we present a map of per capita intake of calories for West Africa. Figure 2 confirms that policy relevant information can be obtained in this way, here highlighting the North-South division in consumption levels in this region, with high consumption along the coast and lower levels in the North, and the higher consumption in urban areas (viz. Ouagadougou and Yamoussoukro).

**Figure 3:**
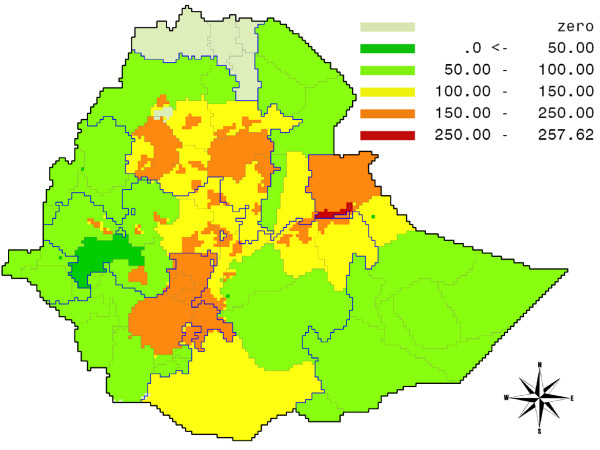
**Hunger gap map for Ethiopia: per capita daily shortfall in food intake, measured in kilocalories**. Source: own calculations.

In analogy with the poverty gap, we can now also compute the hunger gap, as the difference between a normative calorie intake, based on a norm weight (18.5 times the square of the height) and a minimum activity level (the PAL factor is set at 1.58), and the actual calorie intake. The map for Ethiopia in Figure 3 shows that in per capita terms hunger is deepest in Shenile district of Somali Province, in large areas of the Southern Nations and Nationalities Province, and in parts of Amhara, with per capita deficits running between 200 and 257 kcal/day.

## Discussion

### Principal findings

The principal findings of the study are that anthropometric data provide a solid starting point for direct estimates of the prevalence of undernutrition and daily per capita calorie intake of people in SSA. We find that, overall, 17.3% of the population suffers from undernutrition, while per capita calorie daily calorie intake is 2098 Kcal, which by international standards, should be enough to allow for a healthy life on average. However, the geographical spreading (within SSA, but even within countries) of the prevalence of undernutrition and per capita calorie intake is substantial.

### Strengths and weaknesses of the study

The major strength of the study is its use of direct, anthropometric, measures for the assessment of calorie intake and nutritional status, which avoids the often criticized elaborate procedure followed by e.g. FAO [6]. In addition, our estimates are at sub-national level, which allows the identification of hot-spots for policy intervention.

However, there are also some potential weaknesses in the study that need to be addressed. First, the results rely heavily on the DHS data, so a valid question is whether these data are representative and reliable. Secondly, since the DHS data do not cover the whole population, inference rules for the missing groups had to be applied, and this may lead to errors in estimations for the population as a whole. Thirdly, the influence of high child mortality and illness on the results has to be addressed, since surveys obviously only cover those alive, and may not include those that are ill. Finally, especially in SSA, the nutritional status of people may also be highly influenced by the persistent occurrence of crises that lead to migration of large numbers of people as refugees and internally displaced persons (IDPs) and the inflow of food aid in response to these crises. All these points are discussed here.

#### How representative are nutrition surveys?

To assess the quality of the DHS surveys, we compare these measurements with those of other independently collected surveys, implemented within the same country or region, during a time period close to or overlapping with the DHS survey.

Such comparison is only possible for anthropometric surveys on children that have been conducted for much longer in many developing countries. For example, since the mid 1990s UNICEF has been implementing, in cooperation with national organisations, the MICS-surveys (Multi Indicator Cluster Surveys), in which also anthropometric data on children are being collected [47]. The MICS-surveys resemble those of DHS, but they are generally conducted in full separation from the DHS activities. In some countries, national nutrition surveys are executed often at regular time intervals and sometimes as part of a continuous nutrition monitoring system. Height-for-age is the anthropometric indicator used for this purpose because, unlike weight-for-age and weight-for-height, it is not immediately affected by short-run changes in nutritional conditions.

We only compare countries where anthropometric data are available from surveys three years apart at most and preferably with shorter time spans between the surveys. The prevalence rate of children with a low height-for-age is generally reported for either under-five children or under-three children. Hence, when the time span between two surveys is no more than two or three years, a fraction of the targeted population segment (children under five years) will appear in both surveys (albeit through different individuals since they are not panel surveys), as children who were one or two years old at the time of the first survey will be three or four years old at the time of the second survey. It is partly for this reason that, in particular for the anthropometric indicator height-for-age, differences between successive surveys can be expected to remain relatively small. Only over longer periods of time, and in regions where undernutrition is widely prevalent, hopefully reductions, significant changes are foreseen to occur. For the comparisons of successive nutrition surveys, in most cases data were available for the same age groups (e.g. two surveys with prevalence of low height-for-age in children under three years, or two surveys with prevalence of low height-for-age in children under five years). For those countries where in two subsequent surveys the reported prevalence of low height-for-age was reported for different age groups (e.g. in the first survey for children under three, and in the second survey for children under five), a correction factor was used, under the assumption that, on average, the prevalence of low height-for-age in children under five years is 1.15 times higher than the prevalence of low height-for-age in children under three years (own calculations). We now report on the comparison in two figures.

**Figure 4:**
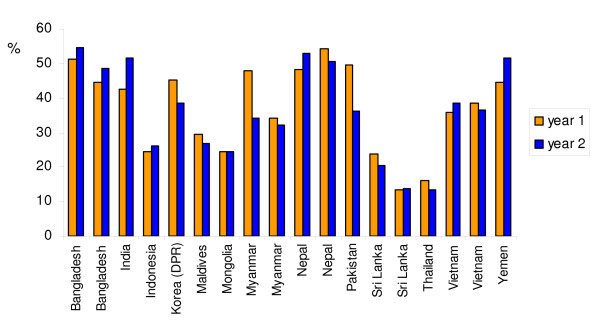
**Undernutrition prevalence rates in children (low height-for-age) in sets of two successive surveys for 13 Asian countries (18 comparison sets)**. Source: own calculations.

Figure 4 compares nutrition surveys held within time spans of generally 2–3 years in 13 Asian countries, with for five countries (Bangladesh, Myanmar, Nepal, Sri Lanka and Vietnam) two sets of data. For more complete information on the years of the surveys, the sample sizes, and whether the data are based on DHS-surveys, MICS-surveys, or derived form other sources [see Additional file 2]. The figure shows that out of the 18 comparison sets, in 13 cases the difference between the two survey results is less than 5 percentage points, and in three cases between 5 and 10 percentage points. Only for Pakistan and for one comparison set of Myanmar, the differences between the two survey results exceed 10 percentage points.

**Figure 5:**
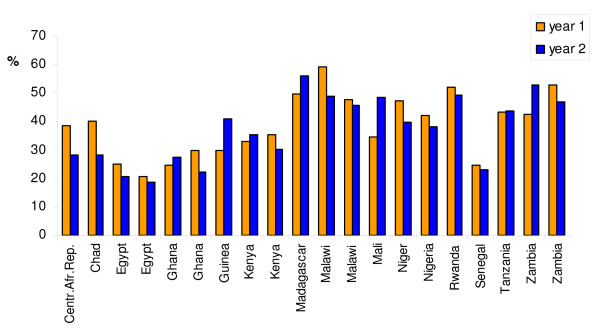
**Undernutrition prevalence rates in children (low height-for-age) in sets of successive surveys for 15 African countries**. Source: own calculations.

Figure 5 compares surveys for 15 African countries [see Additional file 2]. As there are many more DHS-surveys available for African countries than for Asian countries, we limit attention to comparison of cases for which two consecutive DHS-surveys are available or a DHS survey and a non-DHS survey. Here also it appears that for most countries the prevalence rates of low height-for-age as measured in two successive surveys are in reasonable agreement. In eleven comparison sets differences are less than 5 percentage points and in three comparison sets differences between 5 and 10 percentage points. In six cases the agreement between the two successive surveys is much less satisfactory, with differences larger than 10 percentage points. Though these differences might, in principle, reflect actual changes in nutritional conditions, it is more likely that sampling procedures (or even measurement techniques and quality) differed strongly between the two surveys.

**Figure 6:**
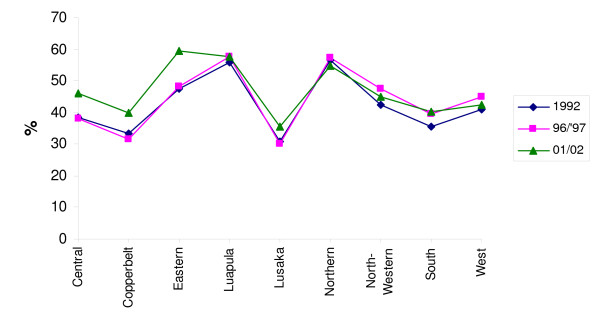
**Undernutrition prevalence rates in children (low height-for-age) in the 9 provinces of Zambia, as reported in three successive surveys over a period of 10 years**. Source: own calculations.

Finally, Figure 6 shows for one country, Zambia, the results of three successive nutrition surveys over a time span of 10 years (1992–2002), hence covering a full change in the cohorts of children surveyed. It appears that also over this longer period of time, the pattern of underweight distribution as measured by height-for-age remains strikingly constant, with only the 2001/2002 results for the regions Central, Copperbelt, and Eastern showing some deviation from the overall pattern.

The comparisons presented in the results section indicate that nutrition surveys held within relatively short time spans tend to remain in reasonable agreement. This applies to surveys implemented by one and the same agency, such as DHS, and in most cases also to surveys that were designed and implemented by different organisations. We also remark that data on child malnutrition as reported in publications by international organizations such as WHO, and UNICEF are, particularly for African countries, largely based on results from DHS surveys, and that these data currently provide the only yardstick actually used for measuring progress in the hunger reduction target under MDG1 [4,26].

Therefore, we conclude that the DHS surveys, which form an important part of the data sources used in the comparisons made in our study, are widely accepted as a reliable data source and yield results that are generally reliable and representative at country level. Furthermore, we have illustrated that they are likely to provide adequate measures at sub-national level as well.

#### Inference rules for missing population groups

Because of the limited availability of the anthropometric data for adult men, and women in some age groups, such as the adolescents and the elderly, actual prevalence rates of undernutrition in these groups might be different and possibly higher than our current estimates, notably for men, children aged 5–9, youth aged 10–14 and elderly above 60. Therefore, we also conducted calculations of the total number of undernourished under adverse assumptions on the prevalence of undernutrition in these groups.

Since the elderly (above 60) on average only comprise between 3.5 and 6 percent of the population, varying assumptions on this age group have a very limited effect on the aggregate: even if we assume that the BMI for this age group (now estimated to be 22.3) was as much as 1.5 points lower on average, the increase in the number of people classified as being undernourished would only shift by 5.2 million, (an increase of 0.7 percent points of the percentage undernourished in total population).

For children aged 5–9, we recall that we have taken the prevalence of underweight children to equal that of the group aged 0–5. For the total group of children (0–9), this implies that 54 million children are undernourished, of which a share of 36% is between 5 and 9 years of age. Even if we assume that the prevalence of underweight children is 25% higher than in the age group 0–5, this only leads to an increase of 5 million children (again an increase of 0.7 percent points of the percentage undernourished in total population).

For the age group of 10–14, we assumed that the nutritional status is equal to that of the group 15–19. Potentially, classification errors for the age group 10–14 could have large effects, since this group comprises on average some 12.5 percent of the total population. Here we can use additional information that is available on the distribution of BMI over the population to assess the number of people that would be classified as being undernourished if the average BMI of this group would be lower than under the current assumptions made. If we assume that the BMI is overestimated by 0.5, 1.0, or 1.5 points, the total number of people classified as suffering from undernutrition increases by 3.8 million people (0.5 percent point), 7.6 million people (1 percent point), and 11.3 million people (1.5 percent point), respectively.

For men, the only available data are for Swaziland and South Africa, where the difference between the percentage of adult males and females with a BMI lower than 18.5 is 7.5 percentage points. If we assume that also for West, Eastern and Central Africa, this is the true distribution of undernutrition by gender, then this would add another 17.9 million undernourished men (2.4 percent point) to our estimate.

Summarizing, our sensitivity checks indicate that even if for adult men and all three age groups we have severely underestimated the prevalence of undernutrition, the total percentage of people suffering from undernutrition (22.5%) would still be 5.3 percent points lower than the FAO estimate of 27.8% [6]. Since the population composition does not differ significantly between the four regions in sub-Saharan Africa, it is very unlikely that even severe errors for specific groups would lead to changes in the regional pattern of undernutrition and consumption.

#### Child mortality and illness

It is a very cold fact that only those children (and adults) that are alive can be weighed and measured. The high under-five mortality in SSA in 2006 (estimated by UNICEF at 160 per 1000 live births) compares very unfavourably with UNICEF estimates of other regions (the second-highest after SSA being South Asia with 83 per 1000 live births) [48]. This could be one explanation for the relatively low prevalence of underweight under-fives in SSA: whereas in South Asia, the prevalence of underweight children in 2006 is estimated at 42%, for SSA this is only 28%. The many endemic diseases, poor quality of drinking water, stronger climatic variability leading to crop failures, possibly also different quality or accessibility of health services are often mentioned explanations for the fact that weaker children will not survive in SSA and will, therefore, not show up in the statistics on underweight children.

Given the wide spread of illnesses among children and adults in Africa, questions may be asked about the effect of these diseases on estimates of undernutrition and food intake. Several effects have to be considered here. First, it may be noted that a reduced food intake due to illness may also cause weight loss in children and adults, and hence, in principle, our method accounts for these losses. However, it may well be that the ill are underrepresented in the survey, causing an underestimate of the number of people suffering from undernutrition. Secondly, illness is a factor that might affect food intake, and effect of these diseases on total food intake should also be taken into account. Among nutritionists, consensus is emerging that the energy requirements of sick patients are usually similar to or lower than those of healthy subjects [49]. The reason is that possible increased metabolic calorie requirements caused by the disease are generally more than offset by a reduced level of physical activity.

A very crude quantitative estimate of a possible reduced level of food intake, for example as a result of the aids epidemic, can be made on the basis of (few) published results on food energy consumption and PAL-levels of aids-patients [50]. According to a review by Elia, PAL-values of patients with HIV/AIDS vary between 1.3 and 1.7, depending on the phase of the disease, with an average PAL-value of approximately 1.5 [51]. Yet, the effect of this reduced PAL-value on total kcal consumption at population level will still be rather limited. For the PAL-value of the healthy segment of the population estimated at 1.8, and assuming that 10% of the population has a reduced PAL of 1.5 as a result of illness, the total resulting average PAL will be 1.77, which is a reduction with about 1.5%.

#### Refugees and food aid

A factor that is not represented in the data used is the persistent refugee problem in SSA, since these people are not captured by the anthropometric surveys discussed [52]. For the year 2005, UNHCR estimated the total number of people of concern to the UNHCR in SSA to be around 5.2 million (or 0.7% of the total population), some 1.7 million of which are refugees in camps who are largely dependent on food aid [53]. Compared to the total population of concern to UNHCR in Asia (8.9 million or 0.02% of total population), it is clear that the impact of missing refugee data is potentially much more severe in Africa than in other regions. Scattered information on the nutritional status of refugees and IDPs in camps is available, mainly from the Food Security Analysis Unit of WFP, but these data depend very much on the time of collection [54]. Secondly, no reliable data is available on the nutritional status of refugees and IDPs outside camps.

Refugees and IDPs in camps are almost fully dependent on the inflow of food aid, but food aid is also distributed to other people in need. Food aid delivery by foreign donors to SSA is substantial: in 2005, roughly 4.6 million tons of cereal equivalents were distributed within the region [55]. On a full ration basis, this is equivalent to 18 million people, or 2.4% of the population, or, if on average all beneficiaries receive 1000 kcal/day, to 38 million people (5% of total population). The effectiveness of the food aid in reducing the observed level of undernutrition remains an open question that is subject of much debate [55-58].

Given the lack of data, we have assumed that for the refugees and IDPs outside camps, the nutritional status is the same as that of the population in the host district, while for refugees and IDPs in camps, we assume that a minimum level of calorie intake of 1800 Kcal per person per day on average can be maintained.

### Comparison with other studies

Table 2 provides the estimates of undernutrition prevalence for the four subregions and the total, and also shows the estimates for 5 individual countries for which estimates from other sources are available for comparison. As can be seen in Table 2, FAO estimates that in SSA as a whole, 210 million people (27.8% of total population) were undernourished in the years 2003–2005 [6]. This figure is obtained starting from the per capita availability of calories for consumption of the Food Balance Sheets, and assuming a distribution of the available calories over the population based on household budget surveys [59,60]. IFPRI estimates, based on Household Expenditure Surveys, are available only for selected countries [10]. In all cases, the estimates are even higher than those of FAO. However, WFP estimates based on independent surveys, come very close to our figures [61-63].

**Table 2:** number of undernourished in millions and percentages of total population in 2005, sub-Saharan Africa, regional totals and selected countries

***Region***	*Own estimates*	*FAO *[6]*	*IFPRI *[10]**	*WFP *[44-46]***
Western Africa	43 (16.3%)	34 (12.9%)		
Central Africa	20 (18.2%)	61 (55.7%)		
Eastern Africa	65 (19.6%)	114 (34.4%)		
Southern Africa	3 (5.9%)	1 (2.0%)		
Total sub-Saharan	131 (17.3%)	210 (27.8%)		

**Ethiopia**	**22 (30.0%)**	**35 (47.7%)**	**56 (76.4%)**	
**Senegal**	**1 (12.2%)**	**3 (36.6%)**	**5 (61.0%)**	
**Tanzania**	**5 (13.0%)**	**13 (33.8%)**	**17 (44.2%)**	**4 (10.4%)**
**Uganda**	**4 (15.2%)**	**4 (15.2%)**	**10 (37.9%)**	**3–4(11.4–15.2%)**
**Niger**	**3 (23.6%)**	**4 (31.5%)**		**4 (31.5%)**

*Country totals aggregated to match region definition, **Percentages referring to 1999 (Ethiopia, Uganda), 2000 (Tanzania) and 2001 (Senegal) are applied to 2005 population totals, ***percentages are applied to 2005 population totals

Table 3 shows our estimates of calorie availability for the four regions, sub-Saharan Africa as a whole and four selected countries for which IFPRI estimates are available [10]. For SSA as a whole, our estimates are only 5 percent above those of FAO [6]. However, comparison at regional level shows remarkable differences. In line with our estimates of undernutrition, the situation seems to be much less dramatic, especially in Central and East Africa, than if the FAO figures are used. In contrast, FAO's very high figures of calorie consumption for West and Southern Africa are not matched by our estimates. On the whole, DHS-based estimates present a picture of Africa that is less diverse geographically. At the country level, a mixed picture emerges in comparing our estimates with those of FAO and IFPRI [6,10]. For Ethiopia and Senegal, our estimates are well above those of FAO and IFPRI, while for Tanzania and Uganda, our estimates are higher than FAO, but lower than IFPRI.

**Table 3:** Per capita availability of calories in 2005, sub-Saharan Africa, regional totals and selected countries

*Region*	Own estimates*	*FAO *[6]**	*IFPRI*[10]***
Western Africa	2321	2518	
Central Africa	2252	1760	
Eastern Africa	2245	1951	
Southern Africa	2618	2858	
Total sub-Saharan	2297	2184	
**Ethiopia**	**2046**	**1810**	**1592**
**Senegal**	**2400**	**2150**	**1967**
**Tanzania**	**2328**	**2010**	**2454**
**Uganda**	**2399**	**2380**	**2636**

*Estimates of intake corrected for losses.**Country totals aggregated to match region definitions. ***data refer to 1999 (Ethiopia, Uganda), 2000 (Tanzania), 2001 (Senegal)

### Policy implications

#### Implications for Africa's record: from nutrition to economics

Our estimates of undernutrition and calorie consumption project a picture of sub-Saharan Africa in 2005 that is substantially less negative than the one generally emerging from reports by FAO and other international agencies. This result is, of course, important in its own right, since it implies that, on average, the number of undernourished is at least 40 million lower than is usually assumed. However, revision of this estimate also has implications for the estimation of economic growth in sub-Saharan Africa in the recent past. The present section takes a glance at these possible implications.

First, the hypothesis can be rejected that the situation in 2005 was exceptionally favourable or achieved after a major breakthrough. Using the same methodology as described above and performing the complete exercise for the year 2000, we find for that year an average calorie intake per capita of 2043 kcal for SSA as a whole, against 2102 in 2005. This improvement in average consumption is largely due to the sharp increase in per capita consumption in Central Africa (up from 1857 to 2052), because of the recovery of Rwanda and Burundi after the end of the war. Hence, the findings for 2005 would indicate that overall food production has kept up with population growth more or less in all regions during the past decade. This would imply that FAO estimates tend to underrate not only the level but also the growth rate of agricultural output achieved in the Central and Eastern regions of Africa.

Second, the outcomes have significant implications for growth outside agriculture as well, especially given the fact that the share of urban population has been rising strongly over the past twenty years. In the period 1980–2005, the share of the urban population increased from 24% to 35% for SSA as a whole. For West, East, Central and Southern Africa, this share increased from 27% to 42%, 15% to 22%, 29% to 40%, and 45% to 56%, respectively [42]. In addition, on average, the per capita calorie intake is around 3% higher than the rural one, where it has to be noted that as regards differences in average per capita kcal intake in urban and rural settings, literature is not unequivocal, with some studies reporting higher kcal consumption in rural areas and other studies reporting higher kcal consumption in urban areas [64,65]. The growing urban population apparently was by and large producing sufficient value added to purchase the implied volumes of consumption.

We therefore conclude that rural areas must have produced a significant and growing agricultural surplus and, barring significant deterioration in agricultural versus non-agricultural terms of trade, that non-agricultural output must have risen as well, to pay for this consumption.

#### Fast updating: the effects of the 2007–2008 food crisis on Ethiopia

Clearly, our estimates of calorie intake for 2005 might be somewhat outdated, particularly because in 2007/2008 a food crisis occurred. More generally, it uses anthropometric data that are not collected every year throughout the continent. Monitoring of progress made in combating hunger through targeted interventions and general policies needs regular intermediate updates. To this effect, we supplement the methodology with a fast estimate that updates the 2005 information, based on more limited recent data, and provide as an illustration an assessment of the impacts of the food crisis in 2008 for Ethiopia.

Despite widespread concern about the effects of the crisis, the magnitude of the impacts still is a topic of controversy. On the basis of field missions, WFP estimates the total number of people in Ethiopia that are affected at 12.5 million, of which 9.6 million are actually being targeted as food aid recipients [66]. Besides price rises, WFP also lists drought, unrest, and animal diseases as causes of increased malnutrition.

In their worldwide assessment of impacts from the food crisis, Ivanic and Martin impute the effects of the rising food prices on the real incomes of people, from which they derive the total number of people that have been pushed below the poverty line, the widely quoted number of 100 million people [67]. This estimate is based on a nine-country household survey (Bolivia, Cambodia, Madagascar, Malawi, Nicaragua, Pakistan, Peru, Vietnam, and Zambia.) for which data on consumption and production of the main food commodities are available. Simulations of food price changes between 2005 – 2007 are carried out for the countries in the sample (0% for beef, 90% for dairy, 80% for maize, 15% for poultry, 25% for rice, 0% for sugar and 70% for wheat), with and without an included effect on wages. The average increase in poverty headcount found in the sample (4.5%) is then applied to all low-income countries to arrive at an increase in poverty count of 105 million people.

Here we simulate the effects of food price increases within a spatial context, to identify the geographic distribution of the "new poor". Our application is for Ethiopia during the period December 2007 – July 2008, based on available data at province level that provide information for a fast update of the nutritional status. Our fast update procedure operates under the following assumptions. First, we choose the province level consumption of 2005 as reference, and assume that until the crisis broke out, that is during 2005–2007, the total per capita calorie intake has remained roughly constant. Secondly, we calculate the budget share of food in total expenditures at province level in December 2007 from the Central Statistical Agency of Ethiopia, and also use food price data at province level to impute the price increase of food for the period January 2008 – July 2008 [68]. Table 4 shows these data by province.

**Table 4:** Food budget shares and price increases of food for Ethiopian regions

	*Budget share food*	*Price increase (%)*
		*Jan – July '08*
Addis Ababa	0.41	69.4
Afar	0.54	57.0
Amhara	0.62	54.4
Benishangul Gumuz	0.57	63.8
Dire Dawa	0.51	69.1
Gambela	0.55	49.6
Oromia	0.59	93.2
SNNP*	0.57	68.5
Somalia	0.61	74.4
Tigray	0.58	79.1
Ethiopia total	0.54	69.4

*Southern Nations, Nationalities and Peoples region. Source: own calculations from CSA (2008)

Using the information on price increases for the period January – July 2008 (second column of table 4), and supposing that for short periods of time, populations can spend almost their whole budget on food (95%), we calculate the maximum daily ration of calories that can be purchased in July, 2008. If this daily ration is lower than the daily calorie intake of December 2007, we obtain a calorie gap.

**Figure 7:**
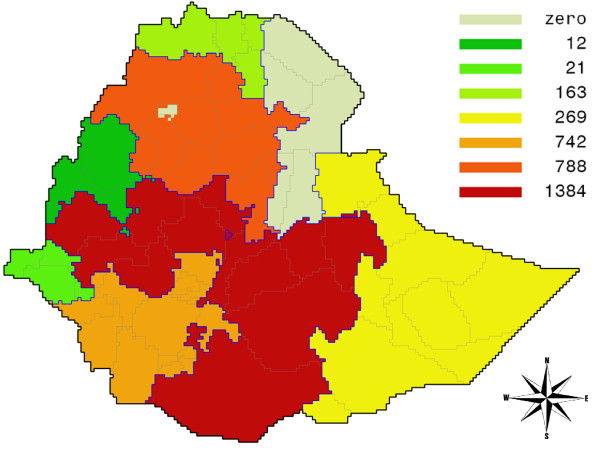
**Ethiopia: Calorie gap for Ethiopia: total shortfall in food intake in billions of Kcal per year per province**. Source: own calculations.

Under these assumptions, only Addis Ababa, and the states of Afar, and Dire Dawa succeed in maintaining their pre-crisis consumption levels. In Gambela, the situation is the most dramatic, with average consumption falling 11%, compared to an average decrease for Ethiopia of almost 7%. For the whole of Ethiopia, the food gap equals almost 3400 billion Kcal on a yearly basis, which is equivalent to 5.14 million Ethiopians on a full ration, the majority of which are concentrated in the Oromia and SNNP (Southern Nations, Nationalities and Peoples) regions. Indeed, these two regions are also named as the two concentration areas for relief food assistance by WFP, next to the Somali region, which ranks fourth in terms of the consumption gap [66]. With respect to its magnitude this number comes close to the WFP estimate of 6.4 million needing immediate food relief, with the qualification that our estimate only accounts for the adverse effects of higher food prices and does not include other important issues, such as the increased prices of fuel [66]. Figure 7 shows the spatial pattern of the calorie gap in billions of Kcal per year within Ethiopia.

### Issues outstanding and future research

Reduction of the number of inference rules needed is, of course, one of the major issues that would improve the quality of the estimates presented in this paper. In particular, the independent measurement of men's weights and heights would be an important addition to the current dataset and would improve the use of the database as a monitoring tool for progress made in combating hunger. Yet, however relevant in the monitoring process, we must acknowledge that even the best of indicators does not shed much light on the underlying causes of hunger and poverty, and the risks that threaten vulnerable populations. This requires further work, including the development of a more comprehensive database of the food economy, in which our anthropometrically-based calculation of calorie intake enter as important input, to be confronted with economic, geo-physical and climatic data within a consistent, spatially explicit database. With this it becomes possible to monitor trends over the medium term. In parallel, case study simulations for selected areas could be conducted, based on limited data sets, to provide fast updates, as illustrated in our small exercise on the impact on Ethiopia of the 2008 price hike.

## Conclusion

As poverty and hunger are basic yardsticks of underdevelopment and destitution, the need for reliable statistics in this domain is self evident. However, while the measurement of poverty through surveys is relatively well documented in the literature and a reasonable degree of methodological consensus has been emerging, for undernutrition, information is much scarcer, particularly regarding adults, and very different methodologies are applied for children and adults. Remarkably, reports on progress made with respect to the Millennium Development Goals published since 2007 no longer mention total undernutrition indicator, not even in their statistical appendices, although FAO provided updates of this indicator for almost all countries in the world for the years 2003–2005, [6]. Consequently, all progress made in terms of combating hunger is now monitored only on the basis of the indicator on children's weights [69,70].

Our paper takes the view that it remains important to include also the nutritional status of adults in assessing progress made in combating hunger. Hence, we use direct anthropometric data on women's weights and BMI in DHS surveys – the same surveys that are used for the assessment of the children – to provide estimates for undernutrition and calorie intake among the total population in sub-Saharan Africa, and assess its reliability. We conclude that our estimates for adults are as reliable as those for children, which are widely and confidently used, and hence, we recommend that more and better use should be made of anthropometric data for adults in estimates of undernutrition world-wide.

## Competing interests

The authors declare that they have no competing interests.

## Authors' contributions

All authors contributed equally in developing this research activity, actual data work and manuscript writing was mainly done by CFAW. All authors have read and approved the final manuscript.

## Supplementary Material

Additional file 1 **Appendix A: data availability and inference rules**. The data provided show the availability of survey data and the use of inference rules for missing data. Click here for file

Additional file 2 **Appendix B: comparison of nutrition surveys**. The data provided show the results of the comparison of nutrition surveys for Asian and African countries. Click here for file
