# Supplementary material for: Estimation of undernutrition and mean calorie intake in Africa: methodology, findings and implications
Source: Int J Health Geogr. 2009 Jun 27;8:37. doi: 10.1186/1476-072X-8-37 (PMC2710326; doi:10.1186/1476-072X-8-37)
Supplement: Additional file 1 — Appendix A: data availability and inference rules. The data provided show the availability of survey data and the use of inference rules for missing data. [file 1476-072X-8-37-S1.pdf]

Appendix A: data availability and inference rules

A.1 Weights of women and children, heights of women

Country	Source*	Year	Comment
Angola	WHO database	2001	Children
Angola	WFP (2005a)	2005	Adults
Benin	DHS	2006	
Botswana	WHO database	2000	Children
Burkina Faso	DHS	2003	
Burundi	DHS	2000	
Cameroon	DHS	2004	
Central African Republic	DHS	1994/1995	
Chad	DHS	2004	
Comoros	DHS	1996	
Côte d'Ivoire	DHS	1999	
Congo, rep.	DHS	2005	
Dem. Rep. Congo	DHS	2007	
Djibouti	MICS	2006	Children
Eq Guinea	WHO	2000	Children
Eritrea	DHS	2003	
Ethiopia	DHS	2005	
Gabon	DHS	2000	
Gambia	MICS	2005	Children
Ghana	DHS	2003	
Guinea	DHS	2005	
Guinea Bissau	MICS	2006	Children
Kenya	DHS	2003	
Lesotho	DHS	2004	
Liberia	WHO database	2000	Children
Madagascar	DHS	2003/2004	
Malawi	DHS	2004	
Mali	DHS	2006	
Mauretania	DHS	2000/2001	
Mauritius	WHO	1995	Children
Mozambique	DHS	2003	
Namibia	DHS	2000	
Niger	DHS	2006	
Nigeria	DHS	2003	
Rwanda	DHS	2005	
Sao Tome	WHO database	2007	Children
Senegal	DHS	2005	
Sierra Leone	MICS	2005	Children
Somalia	MICS	2006	Children
South Africa	WHO	1999	Children
South Africa	DHS	1998	Women and men
Sudan	WHO database	2000	Children
Swaziland	DHS	2006	Women and men
Tanzania	DHS	2005	
Togo	DHS	1998	
Uganda	DHS	2006	
Zambia	DHS	2001/2002	
Zimbabwe	DHS	2005	

* notes: DHS = Demographic and Health Surveys, USAID, MICS=Multi Indicator Cluster Surveys, UNICEF

A.2 Inference rules applied

Imputed country	*Imputed variable*	*Reference country*	*Remarks*
Botswana	Adult calorie intake	Namibia	Corrected for child calorie intake
Burundi	Adult calorie intake	Ethiopia	Corrected for child calorie intake
Eq. Guinea	Adult calorie intake	Namibia	Corrected for child calorie intake
Djibouti	Adult calorie intake	Kenya	Corrected for child calorie intake
The Gambia	Adult calorie intake	Senegal	Corrected for child calorie intake
Liberia	Adult calorie intake	Burkina Faso	Liberian child calorie intake used to create spreading of consumption
Mauritius	Adult calorie intake	Gabon	Corrected for child calorie intake
Guinea Bissau	Adult calorie intake	Niger	Corrected for child calorie intake
Sao Tome	Adult calorie intake	South Africa	Corrected for child calorie intake
Sierra Leone	Adult calorie intake	Niger	Sierra Leonean child calorie intake used to create spreading of consumption
Somalia	Adult calorie intake	Ethiopia	Corrected for child calorie intake
Sudan	Adult calorie intake	Ethiopia	Corrected for child calorie intake, adjustment for Darfur
Cape Verde	Adult and child calorie intake	Gabon	
Reunion	Adult and child calories intake	Gabon	
St Helena	Adult and child calorie intake	Gabon	
Seychelles	Adult and child calorie intake	Gabon	
