# Supplementary material for: Estimation of undernutrition and mean calorie intake in Africa: methodology, findings and implications
Source: Int J Health Geogr. 2009 Jun 27;8:37. doi: 10.1186/1476-072X-8-37 (PMC2710326; doi:10.1186/1476-072X-8-37)
Supplement: Additional file 2 — Appendix B: comparison of nutrition surveys. The data provided show the results of the comparison of nutrition surveys for Asian and African countries. [file 1476-072X-8-37-S2.pdf]

Appendix B Comparison of nutrition surveys

Table B1 Comparison of nutrition surveys for Asian countries

Country	Years of surveys	Children with height-for-age <-2sd	Sample-size	Source
Bangladesh	1995-96	51.4	2614	Non-DHS
	1996-97	54.6	4787	DHS
Bangladesh	1999-2000	44.6	5421	DHS
	2001	48.5	71931	Non-DHS
India (rural)	1996-97	42.6	22959	Non-DHS
	1998-99	51.6	24396	DHS
Indonesia (WFA)*	2000	24.6	70602	Non-DHS
	2001	26.1	11693	Non-DHS
Korea (DPR)	2000	45.2	4175	MICS
	2002	38.6	5232	Non DHS
Maldives	1994	29.6	1994	Non DHS
	1995	26.9	798	MICS
Mongolia	1999	24.6	4037	Non DHS
	2000	24.6	5784	MICS
Myanmar*	1997	47.8	4894	Non DHS
	2000	34.2	8081	MICS
	2003	32.2	5390	MICS
Nepal	1996	48.4	3705	DHS
	1996	53.1	5525	MICS
	1997/98	54.2	17241	Non DHS
	2001	50.5	6409	DHS
Pakistan	1990-91	49.6	4056	DHS
	1990-94	36.3	3141	Non DHS
Sri Lanka	1993	23.8	3067	(semi)DHS
	1995	20.4	2782	Non DHS
Sri Lanka	2000	13.5	2531	(semi) DHS
	2001	13.9	1716	Non DHS
Thailand	1993	16.0	11748	Non DHS
	1995	13.4	4178	Non DHS
Vietnam	1998	35.9	12919	Non DHS
	1999	38.7	93469	Non DHS
	2000	36.5	94469	Non DHS
Yemen	1996	44.6	3833	MICS
	1997	51.7	7501	DHS

*: Corrections made to extrapolate undernutrition in children <3 years to children <5 years, by multiplying percentage children with low height-for-age with factor 1.15; Sources: DHS, WHO

Table B2 Comparison of nutrition surveys for African countries

Country	Years of surveys	Children with height-for-age <-2sd	Sample size	Survey type/ organisation
CAR*	1994/95	38.6	2310	DHS
	1995	28.4	2225	Non DHS
Chad	1996/97	40.1	5665	DHS
	2000	28.1	5043	Non DHS
Egypt	1997-98	24.9	3328	DHS
	1998	20.6	3997	DHS
	2000	18.7	10193	DHS
Ghana	1987-88	24.6	1492	Non DHS
	1988	27.6	2011	DHS
	2003	29.9	3183	DHS
	2006	22.4	3166	MICS
Guinea	1999	29.9	2452	DHS
	2000	40.9	1457	Non DHS
Kenya	1998	33.0	4413	DHS
	2000	35.3	5917	MICS
	2003	30.3	5306	DHS
Madagascar*	1995	49.8	5049	MICS
	1997	53.1	3080	DHS
Malawi	1997-98	59.1	6309	Non-DHS
	2000	49.0	9322	DHS
	2004	47.8	8520	DHS
	2006	45.9	20747	MICS
Mali*	1995-96	34.6	4678	DHS
	1996	48.6	n.a.	MICS
Niger*	1998	47.3	4022	DHS
	2000	39.7	4616	MICS
Nigeria	2001	42.0	4954	Non DHS
	2003	38.3	4789	DHS
Rwanda (rural)	1991-92	52.2	1939	Non DHS
	1992	49.4	4177	DHS
Senegal	1992-93	24.7	3865	DHS
	1996	22.9	n.a.	MICS
Tanzania	1996	43.4	5344	DHS
	1999	43.8	2821	DHS
Zambia	1996/97 (national)	42.4	5443	DHS
	1999 (national	53.0	1095000	MICS
	2001 (national	46.8	5784	DHS

*: Corrections made to extrapolate undernutrition in children <3 years to children <5 years, by multiplying percentage children with low height-for-age with factor 1.15; Sources: DHS, WHO
